# Upper eyelid contour symmetry measurement with Bézier curves

**DOI:** 10.5935/0004-2749.20200002

**Published:** 2020

**Authors:** Marcelo Blochtein Golbert, Denny Marcos Garcia, Patrícia Mitiki Santello Akaishi, Antonio Augusto Velasco e Cruz

**Affiliations:** 1 Department of Ophthalmology, School of Medicine of Ribeirão Preto, Universidade de São Paulo, Ribeirão Preto, SP, Brazil; 2 Craniofacial Research Support Center, Universidade de São Paulo, São Paulo, SP, Brazil

**Keywords:** Eyelids/anatomy & histology, Bézier curve, Facial asymmetry/diagnosis, Reference values, Image processing, computer-assisted, Pálpebras/anatomia & histologia, Assimetria facial, Curva de Bézier, Valores de referência, Processamento de imagem assistida por computador

## Abstract

**Purpose:**

The purpose of the present work is to measure the interocular upper lid contour symmetry using a new method of lid contour quantification.

**Methods:**

The Bézier curve tool of the Image J software was used to extract the right and left upper eyelid contours of 75 normal subjects. Inter-observer variability of 29 right lid contours obtained by two independent observers was estimated using the coefficient of overlap of two curves and an analysis of the differences of the contour peak location. A two-way analysis of variance was used to test the mean value of the coefficient of overlap of the right and left contours in males and females and lid segments. The same analysis was performed to compare the location of the contour peak of the right and left contours.

**Results:**

The coefficient of contour overlap obtained by independent observers ranged from 93.5% to 98.8%, with a mean of 96.1% ± 1.6 SD. There was a mean difference of 0.02 mm in the location of the contour peak. Right and left contour symmetry did not differ between females and males and was within the range of the method variability for contour overlap and location of the contour peak.

**Conclusions:**

The upper eyelid contour is highly symmetrical. Bézier lines allow a quick and fast quantification of the lid contour, with a mean inter-observer variability of 3.9%.

## Abstract (pt)

**Objetivo:**

O objetivo do presente estudo é mensurar a simetria interocular do contorno da pálpebra superior por meio de um novo método de quantificação de contorno palpebral com curvas de Bézier.

**Métodos:**

A ferramenta de curva de Bézier do software ImageJ foi utilizada para extrair os contornos palpebrais direito e esquerdo de 75 sujeitos normais. A variabilidade interobservador de 29 contornos palpebrais do olho direito obtidos por dois observadores diferentes foi estimada pelo coeficiente de superposição de duas curvas e pela análise das diferenças das posições do pico do contorno. Análise de variância de dois fatores foi empregada para testar a média do coeficiente de superposição entre os contornos direito e esquerdo quanto ao sexo e segmento palpebral. A mesma análise foi utilizada para comparar a localização do pico do contorno dos olhos direito e esquerdo.

**Resultados:**

O coeficiente de superposição obtidos por observadores independentes variou ente 93,5% e 98,8% com média de 96,1% ± 1,6 DP. A diferença das médias da localização do pico do contorno palpebral foi de 0,02 mm. A simetria entre os contornos dos olhos direito e esquerdo não diferiu entre o sexo feminino e masculino e esteve na faixa de variabilidade do método para o coeficiente de superposição e localização do pico do contorno.

**Conclusões:**

O contorno da pálpebra superior é altamente simétrico. As linhas Bézier permitem uma rápida e prática quantificação do contorno palpebral com uma média de variabilidade interobservador de 3,9%.

## INTRODUCTION

Margin reflex distance (MRD_1_) is the parameter most frequently used to characterize the upper lid position of any type of patient, including those who undergo surgical correction for ptosis or lid retraction^(1)^. However, it is well known that lids with an identical MRD_1_ may have different contours^(2)^. The question then arises of what degree of interocular contour asymmetry is acceptable following a monocular or bilateral upper lid surgery. The answer is largely unknown because there are no published data about the magnitude of lid contour symmetry in the normal population. In the present study, we describe a new method of lid contour measurement based on a semi-automated method of curve adjustment (Bézier curves) to estimate the range of interocular upper lid contour differences in normal subjects. Bézier curves are extremely versatile graphical tools because they are expressed by parametric equations (see the Appendix).

## METHODS

### Subjects

The NIH ImageJ public domain software (available for free download at https://imagej.nih.gov/ij/) was employed to extract the upper eyelid contours of a sample consisting of 75 normal subjects ranging in age from 19 to 76 years (mean= 44.6 ± 15.7 SD). There were 42 females (mean age= 44.5 ± 15.5 SD) and 33 males (mean age= 44.6 ± 16.3 SD). None of them had any systemic disease or had previously undergone lid surgery. All participants were comfortable with the shape and position of their upper lids.

After providing informed consent and receiving institutional review board approval, both eyes of the subjects were photographed in the primary position of gaze. The images were then transferred to a microcomputer and processed using the ImageJ software.

### Lid contour extraction

**Figure 1:**
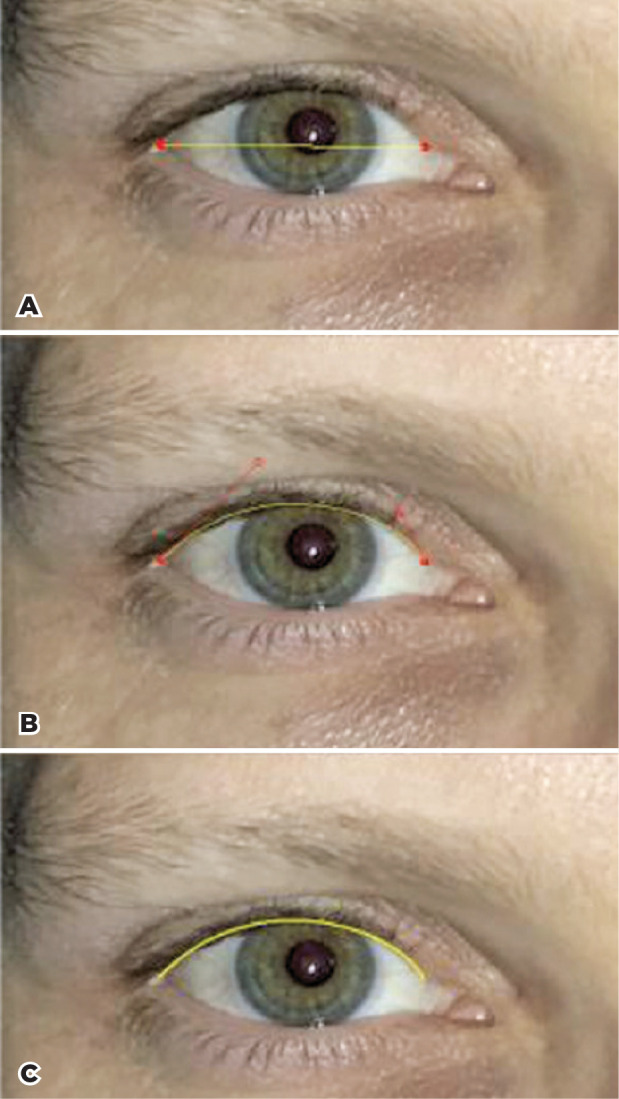
Adjustment of a Bézier line to the lid contour moving freely between two control points (red circles). A) Initial Bézier. B) Line adjusted to the upper contour. C) Line drawn.

**Figure 2:**
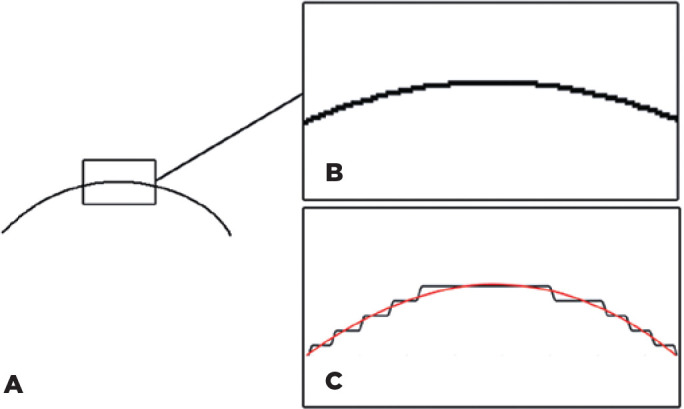
A) Black line after binarization of the yellow Bézier line. B) Upper, small irregularities due to pixel dimension. C) Effect of smoothing the curve using a Savitzky-Golay filter.

Using the Bézier icon of the software, the user clicked on the lateral canthus and on the end of the ciliated portion of the upper lid to create a straight line defined by two control points (Figure 1 top). Then, the user was able to freely modify the straight line to adjust it to the lid contour by clicking on any of the two control points and dragging them around (Figure 1 middle). The resultant Bezier line was draw with a preselected line color (Figure 1 bottom). We used a yellow hue, but any color that has no correspondence with the spectrum of human facial hues might be employed. Finally, the image was binarized (color threshold tool) in such way that only the pixels forming the contour were displayed in black. The remaining pixels were converted to white and not shown (Figure 2).

**Figure 3:**
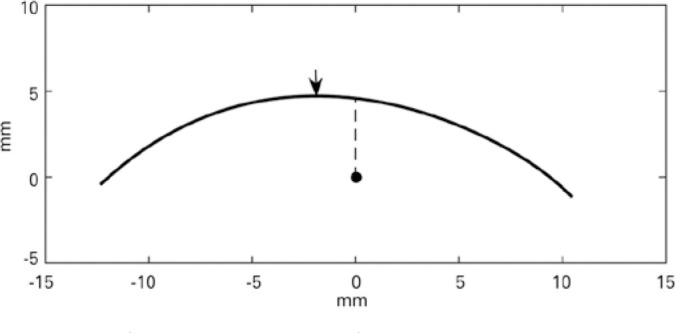
Final characterization of the lid contour: The arrow indicates the contour peak. The dark circle is the pupil center.

The numerical coordinates of the line representing the contour were saved and transferred to a statistical software for graphical analysis (Matlab 8.5, The MathWorks Inc., Natick, MA). The line expressing the lid contour was resampled to contain 1000 points (a spatial resolution of approximately 0.03 mm) and smoothed using a Savitzky-Golay filter. The final contour line relative to the pupil center is displayed in figure 3.

### Test-retest reliability of contour extraction

Two of the authors independently isolated the upper lid contour of a subset of 29 subjects ranging in age from 19 to 73 years (mean= 43.9 ± 16.4 SD). The Bland-Altman^(3)^ plot was employed to test the inter-observer variability of the position of the contour peak determination. This contour peak corresponds to the point where the first derivative is equal to zero.

**Figure 4:**
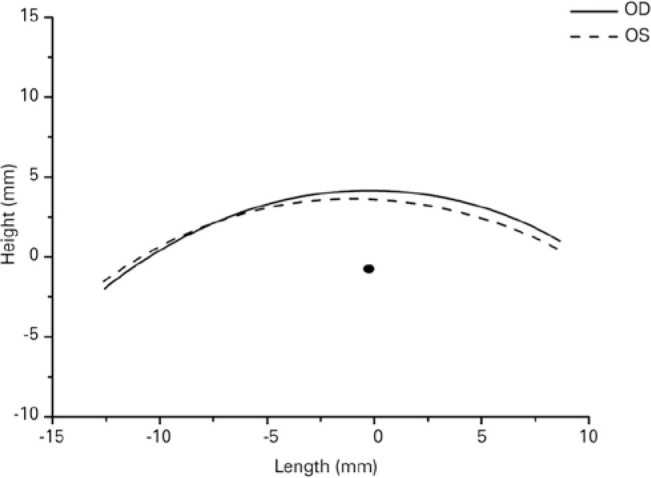
The coefficient of the superposition of two superimposed lines determined for one subject. The software calculates the agreement of the position of each point of the two curves according to the formula: $POC(\%)=\left(1-\frac{\sum_{i=1}^{N}\left|OD_{i}-OS_{i}\right|}{\sum_{i=1}^{N}\left|OD_{i}+OS_{i}\right|}\right)\times 100$

The degree of agreement between the two contours was also estimated by measuring a coefficient of overlap of two curves (POC). To calculate the degree of overlap of the right and left contours, the mid-pupil center point was used to superimpose the two lines (the left contour was flipped to match the lateral and medial portions of the two contours), and the software compared the two lines point-to-point, expressing their agreement as a percentage (Figure 4).

### Interocular contour symmetry

**Figure 5:**
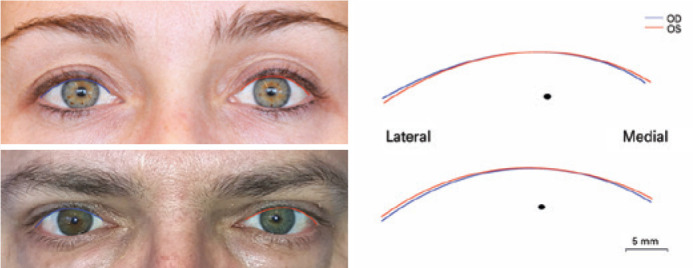
Minute asymmetries between the right and left upper eyelid contours of two subjects extracted with Bézier lines (right). Blue represents the right contour, and red represents the left contour.

The degree of agreement between the right and left lid contours was estimated by measuring the above coefficient of overlap of the two curves. This analysis was performed for the entire lid and for the medial and lateral parts of the lid relative to the pupil center (Figure 5).

To investigate a possible effect of gender on the degree of contour symmetry, two-way analyzes of variance were employed to compare the coefficients of the right and left lid overlap and the right and left locations of the contour peak between females and males. All calcu lations were performed using JMP software version 10.0 (SAS Institute Inc., Cary, NC, USA).

## RESULTS

### Test-retest reliability of contour extraction

**Figure 6:**
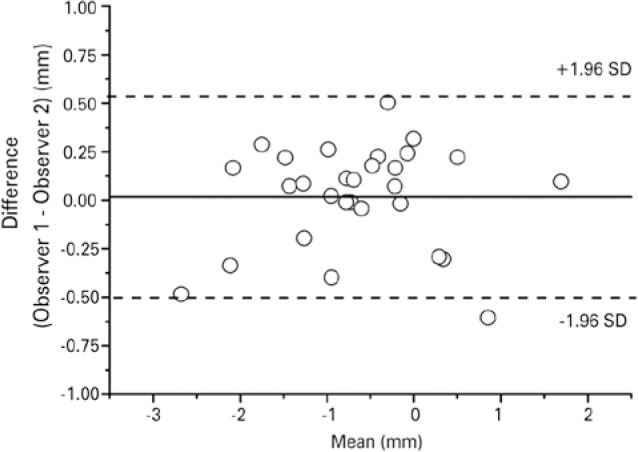
Bland-Altman plot of the differences of the contour peak location of the same lids extracted by two independent observers with Bézier curves.

The coefficient of overlapping of the lid contours determined by the two observers ranged from 93.5% to 98.8%, with a mean of 96.1% ± 1.6% SD. The differences between observers in the position of the peak contour were very small, with a mean value of 0.02. The distribution of these differences ranged from 0.54 to -0.5 mm, as shown by the Bland-Altman plot (Figure 6).

### Contour symmetry in normal subjects

The mean MRD_1_ of the females (OU= 3.4 mm ± 0.11 SE, OS) was slightly higher than the mean MRD_1_ of the males (OU= 2.9 mm ± 0.16 SE), but there were no differences between eyes in either group.

Table 1 lists the mean values of the coefficient of overlap for the right and upper contours and for the lateral and medial segments of the contours relative to the mid-pupil center. A two-way ANOVA indicated that there was no difference between genders (F=2.9, p=0.09) or between lid segments (F=0.25, p=0.61). Moreover, the gender vs lid segment interaction was non-significant (Figure 2). The contour peak was significantly more lateral in females than in males (F=10.16, p=0.002), but there were no differences between eyes in either group (females OD= -0.8 ± 0.11, OS= -0.6 ± 0.12; males OD= -0.25 ± 0.13, OS = -0.18 ± 0.14).

**Table 1:** Degree of agreement of interocular upper lid contours in normal subjects

Lid segment	Gender	
	Females	Males
Lateral	95.0 ± 0.50	93.9 ± 0.70
Medial	94.8 ± 0.48	93.7 ± 0.80
Whole lid	94.9 ± 0.41	93.7 ± 0.56

## DISCUSSION

Since we began addressing the problem of how to extract and mathematically analyze the line formed by the edge of the lid margins and the eyeball^(4)^, a large amount of data has been gathered. The geometry of the parabolic nature of the upper lid contour has been demonstrated^(5)^, the temporal and nasal areas of the fissure have been compared in normal subjects and patients with Graves upper lid retraction^(6)^, and it was recently shown that the lid contour could be extracted with multiple radial mid-pupil distances^(7)^.

Despite this body of knowledge, a precise and flexible graphical method of quantification of the two-dimensional shape of the upper or lower eyelid contour was still lacking. One of the main problems with the methods previously described was the amount of points available to represent a continuous line. Localized distortions are not well displayed if the contour line is not continuous. Likewise, in severe cases of ptosis, it is difficult to extract the contour with multiple radial distances because the central portion of the lid tends to be overrepresented.

Our attention was driven to the Bézier lines after reading a recent investigation that used the Bézier principles to measure the area under the lower lid contours^(8)^. Pierre Étienne Bézier was an engineer who worked as the director of Renault car designers in France. His main line of work was to develop mathematical tools to quantitatively describe the curves and surfaces drawn by car designers. The mathematical principles underlying the Bézier curves are briefly described in Appendix 1, but it is important to recognize that they are now widely used in computer-aided technology^(9^,^10)^.

The Bézier plugin in the NIH ImageJ software is extremely versatile. The two control points are sufficient to adjust a curve to the ciliated portion of the upper eyelid. Other control points can be added to outline more complex curves.

The degree of symmetry of the facial soft tissues is of paramount importance in the evaluation of beauty. However, as pointed out by Ferrario, the craniofacial complex is not a perfectly symmetrical system, and when the right and left halves of the face are superimposed, different degrees of asymmetries are detected^(11)^. This subject is important in oculoplastic procedures because the periocular area is preferentially scanned when age is being judged^(12)^.

Our results indicate that the right and left upper lid contours are highly symmetrical. When they were superimposed, the mean degree of asymmetry was less than 4% for the entire extent of the lid or its segments. We did not notice any differences in the medial and lateral portions of the lid. The magnitude of difference between the lid contours is within the variation between observers and likely reflects methodological issues and is not true asymmetry. Small differences in the position of the two control points of the Bézier plugin (lateral canthus and the end of the ciliated portion of the lid) can lead to slight contour asymmetries.

Oculoplastic surgeons addressing ptosis and upper eyelid retraction must carefully examine the lid contours, with a high grade of symmetry being the optimal result in any type of surgical manipulation of the lid position. We hope that the method that we described here will help surgeons improve the results of lid positional anomalies.

## Appendix 1

In mathematics, parametric equations are a group of complex curves obtained when x and y are defined according to a third variable called a parameter (t). In this type of equation, the x and y coordinates are determined by a pair of different functions. This means that the Bézier lines are mathematically expressed by a pair of equations: one for the x coordinate and the other for the y coordinate.

In the case of a line constructed with three control points, the line is given byIf a fourth control point is added, the resulting line is given by

$$\begin{array}{rl} & x_{C1}=(1-t{)}^{2}x_{0}+2(t-1)tx_{1}+t^{2}x_{2}\\ & y_{C1}=(1-t{)}^{2}y_{0}+2(t-1)ty_{1}+t^{2}y_{2} \end{array}$$

If a fourth control point is added, the resulting line is given by

$$\begin{array}{c} x_{C}=(1-t{)}^{3}x_{0}+3t(1-t{)}^{2}x_{1}+3t^{2}(1-t)x_{2}+t^{3}x_{3}\\ y_{C}=(1-t{)}^{3}y_{0}+3t(1-t{)}^{2}y_{1}+3t^{2}(1-t)y_{2}+t^{3}y_{3} \end{array}$$
